# Nanocurcumin–pyrroloquinoline formulation prevents hypertrophy–induced pathological damage by relieving mitochondrial stress in cardiomyocytes under hypoxic conditions

**DOI:** 10.1038/emm.2017.199

**Published:** 2017-12-01

**Authors:** Sarita Nehra, Varun Bhardwaj, Anju Bansal, Pronobesh Chattopadhyay, Deepika Saraswat

**Affiliations:** 1 Experimental Biology Division, Department of Experimental Biology, Defence Institute of Physiology and Allied Science, Defence Research and Development Organization, Timarpur, New Delhi, India; 2 Defence Research Laboratory, Tezpur, Assam, India

## Abstract

This study investigates the therapeutic effect of a nanocurcumin formulation (NCF) containing nanocurcumin (NC) and pyrroloquinoline quinone (PQQ) on ameliorating hypoxia-induced stress in hypertrophied primary human ventricular cardiomyocytes (HVCM) under hypoxic conditions, as validated in a Sprague-Dawley rat model of chronic hypobaric hypoxia (cHH)-induced right ventricular hypertrophy (RVH). Based on our previous findings, here, we analyzed the improvement in the protective efficacy of NCF against mitochondrial damage. The electron transport chain Complexes’ activities were analyzed as a chief operational center for mitochondrial homeostasis, along with key gene and protein markers for mitochondrial biogenesis, redox function, fatty acid oxidation, bio-energetic deficit and cell survival. NCF supplementation imparts cyto-protection from hypoxia-induced hypertrophy and damage in both *in vitro* and *in vivo* models while maintaining mitochondrial homeostasis better than NC and PQQ alone. This study proposes the use of NCF as a potential candidate molecule for imparting protection from high altitude-induced maladies in ascendants.

## Introduction

Prolonged hypoxia-induced hypertrophy can cause pathological damage in cardiomyocytes that appear in the form of impaired mitochondrial homeostasis.^1, 2, 3, 4^ The optimal functioning of mitochondria is tightly regulated by transcriptional control of the nuclear genome, which encodes mitochondrial regulatory proteins, including those required for operating electron transport chain (*e.t.c.*) Complexes I–V.^5^ However, the functions of these key regulatory proteins and Complexes are compromised under stress and cause cyto-damage by promoting excessive free radical leakage (Complexes I and III),^6^ metabolic imbalance (Complexes II and IV)^7^ and bio-energetic deficit (Complex V).^8^ Studies have shown close associations of impaired *e.t.c.* Complexes in cardiovascular damage.^2, 9^ However, whether hypoxic stress promotes damage to mitochondria in hypertrophied cardiomyocytes remains unclear.

Multiple molecular pathways are involved in the modulation of cardiac hypertrophy. Akt/Gsk-3β-mediated signaling remains the key modulatory pathway, among others. Akt and Gsk-3β are positive and negative regulators of cardiac hypertrophy, respectively.^10^ Akt phosphorylation and activation is directly involved in the modulation of cardiac hypertrophy by controlling downstream signaling cascades.^11^ In contrast, phosphorylation of Gsk-3β promotes its deactivation and further modulates hypertrophy.^12^ Studies have shown that Akt phosphorylation and activation promote de-phosphorylation of Gsk-3β, which in turn promotes cardiac hypertrophy. Many studies have shown that the stress-induced activation of Gsk-3β and the resulting induction of cardiac hypertrophy are deleterious. Further, Gsk-3β activation is directly involved in the regulation of primary transcriptional regulators of hypertrophy, that is, GATA-4.^13^ Similarly, NFκB remains an important redox-sensitive transcriptional regulator that is required to maintain a variety of cellular functions, such as cell growth, survival and inflammation.^14^ The canonical pathway of NFκB signaling (NFκB-p65/RelA) has been well elucidated as a potential regulator of cardiomyocyte hypertrophy.^15^ Studies have shown that NFκB expression is essential for promoting hypertrophic growth in cardiomyocytes.^16^ Knock-out studies relying upon deletion of functions of either functional units or inhibitors of NFκB stabilizers have shown to be lethal in embryonic developmental and can modulate cardiac dysfunction under stress.^17, 18^ In addition to agonists, ligands and endotoxin-mediated onset of NFκB signaling, mitochondrial reactive oxygen species (ROS) are also crucially involved in cellular pathology via the activation of NFκB by regulating programmed cell death.^19, 20, 21^ However, the association between hypoxia-induced NFκB activation and its potential effect on mitochondrial homeostasis in hypertrophied cardiomyocytes remains unknown.

Use of natural bio-active compounds promotes homeostasis under stress with minimal undesirable effects. In this regard, we have previously shown that nanotized curcumin (NC) ameliorates hypoxia stress both *in vitro* and *in vivo.*^3, 4, 22, 23^ Hypoxic stress elicits multiple physiological damages on normal physiological functions.^24^ Additionally, the normal functioning of cardiomyocytes critically relies upon an optimal oxygen supply. Thus, there remains a need for highly potent bio-active therapeutic compounds to abolish hypoxia-induced impairments. Therefore, we hypothesized that a combination of therapeutic bio-active compounds might demonstrate a potential strategy for reducing hypoxia-induced multi-faceted pathological damage. Pyrroloquinoline quinone (PQQ), a natural poly-phenolic, redox-factor and anti-oxidant compound of high nutritional value, is a potential candidate for improving hypoxia-induced damage in cardiomyocytes.^25^ PQQ can regulate biological functions,^26^ including mitochondrial biogenesis,^27^ redox functions^28, 29^ and reproductive health.^30^ Thus, to further improve the therapeutic properties of NC, we propose the use of a nanocurcumin formulation (NCF) consisting of NC and PQQ together as a potential candidate molecule to ameliorate hypoxia-induced stress in hypertrophied cardiomyocytes.

## Materials and methods

Ultra-pure, molecular grade chemicals, including PQQ (D7783), were procured from Sigma Aldrich (St Louis, MO, USA) or as otherwise stated. Antibodies were purchased from SantaCruz Biotechnology, Inc., Dallas, TX, USA, or as otherwise stated. Nanocurcumin (average particle size 200 nm, zeta potential −131 mV) was obtained as a kind gift from Professor Santosh Kar (KIIT University, Odisha, India).

### Study design

The study was designed to prepare, characterize and assess improvements in the protective efficacy of NCF in *in vitro* and *in vivo* models of hypoxia-induced hypertrophy. Here we analyzed the effects of hypoxic stress on changes in mitochondrial regulators of homeostasis and its effects on the functions of *e.t.c.* Complexes. We also assessed the effect of NFKB activation on the molecular regulation of hypoxia-induced hypertrophy. Experimental animals and cell culture studies were performed in eight experimental groups, that is, normoxia vehicle controls (NVC), normoxia plus nanocurcumin (N+NC), normoxia plus PQQ (N+PQQ), normoxia plus nanocurcumin formulation (N+NCF), hypoxia vehicle controls (HVC), hypoxia plus nanocurcumin (H+NC), hypoxia plus PQQ (H+PQQ) and hypoxia plus nanocurcumin formulation (H+NCF). Sterile, neutral phosphate-buffered saline (PBS) was used as vehicle. All results were compared to NC- or PQQ-treated animals to compare improvements in the therapeutic potential of NCF under hypoxic conditions.

### Preparation and characterization of NCF

A detailed description of the preparation and characterization of NCF can be found in the Supplementary Material.

### Cell culture, hypoxia exposure and protein isolation

HVCM cells were maintained under the conditions of normoxia or hypoxia as previously described.^3^ Nuclear and cytoplasmic protein extracts and protein estimations were prepared according to the method described previously.^4^

### *In vitro* uptake and toxicity analysis of NCF

NCF was suspended in sterile PBS, and the suspension was stabilized via ultra-sonication for 15 min at 4 °C (Sonics Vibra Cell, Sonic and Materials, Inc., Newtown, CT, USA, pulse cycle 9 s, amplitude 40%). The cellular uptake of NCF was evaluated according to the method described by Kunwar *et al.*^31^ Briefly, the HVCM cells were grown to confluence in 96-well black plates and incubated with 100 μM of NCF for 24 h. The culture medium was removed, and cells were washed twice with PBS. Spectro-fluorometric analysis (FLUOStar Omega, BMG Labtech, Ortenberg, Germany) was performed to evaluate the cellular uptake of NCF (excitation wavelength/emission wavelength, that is, *λ*_ex_/*λ*_em_ read at 360/420 nm for NC and 360/460 nm for PQQ). An increase in NCF uptake was represented as a percentage change compared with normoxia vehicle control cells.

A detailed description of the *in vitro* cytotoxicity analysis can be found in the Supplementary Material.

### Cellular viability and hypertrophy

A detailed description can be found in the Supplementary Material.

### Analysis of mitochondrial damage under hypoxic conditions in HVCM cells

Hypoxia-induced changes in mitochondrial membrane potential (ψ_m_) were analyzed as indicative of mitochondrial damage using a MitoLight Apoptosis Detection kit (APT242, Millipore, Billerica, MA, USA), and images were acquired (× 40) using a fluorescent microscope (IMAGER.M2, AxioCam MRc5, Carl Zeiss, Oberkochen, Germany). Further, cellular oxidative stress was analyzed in HVCM cells under hypoxic conditions for confirmation of cyto-damage. The mitochondrial redox balance was assessed by evaluating MnSOD (Manganese-superoxide dismutase) activity and ROS leakage as previously described.^4, 32^

### Immunocytofluorescence

A detailed description can be found in the Supplementary Material.

### Experimental animals and ethics statement

Age-matched adult male Sprague-Dawley rats (220±10 g) (6–8 weeks) were used for all of the experiments. The experimental animals were kept in the Institute’s Experimental Animal Facility in clean cages and had equal access to water and standard chow (Lipton India Ltd., West Bengal, India), maintaining a normal 12 h light and dark cycle at 28±2 °C temperature and relative humidity (55±2%). All experiments were approved by the Institutional Animal Ethical Committee in accordance with the Committee for Purpose of Control and Supervision of Experiments on Animals (27/1999/CPCSEA), Government of India.

### *In vivo* toxicity assessment

Sub-acute toxicity in rats supplemented with 2000 mg kg^−1^ per day of NCF, p.o. for 28 days was performed by following the OECD guidelines. The animals were closely monitored for changes in body weight, fur texture and behavior and the development of skin infections. After completion of the experiment, the animals were killed by overdose of ketamine (80 mg kg^−1^ b.w.) and xylazine (10 mg kg^−1^ b.w.) as previously described.^23^ Blood was withdrawn by left-ventricular puncture and was immediately subjected to blood-gas content analysis using an i-STAT analyzer (Abott, IL, USA) and hematological profiling (MS-4 Autoanalyzer, Melet Schloesing Laboratory, Osny, France). Post-drawing blood, animals were fixed in 4 % neutral paraformaldehyde (PFA) for histopathological analyses of liver, heart and lungs.

### Pharmacokinetic assessment of NCF in Sprague-Dawley rats

A detailed description can be found in the Supplementary Material.

### Morphometry analysis and RVH

Morphometry analysis of right ventricular hypertrophy (RVH) was performed by analyzing Fulton’s index (RV/LV+S and RV/BW) and histopathological assessment via Masson’s trichrome staining as previously described.^22^ Circulating levels of atrial natriuretic factor (ANF) (ab108797, Abcam, Cambridge, UK) and brain natriuretic peptide (BNP) (ab108816, Abcam) were estimated as markers of cardiac hypertrophy using commercially available ELISA kits; the assays were performed according to manufacturer’s instructions. Tissue distribution of ANF was examined as a marker of hypertrophy by immunohistochemistry, and changes in tissue-architecture were analyzed by immunofluorescence staining using α-actin/DAPI. Validation of cHH-induced RVH was performed by analyzing the key molecular pathway for regulation of hypertrophy, that is, the Akt/Gsk-3β-mediated signaling cascade.^33^

Changes in pulmonary vascular architecture and morphometry post cHH exposure were made in PFA-fixed lung tissue by H&E staining. Tissue sections were microscopically visualized for increments in pulmonary artery medial wall thickness and changes in pulmonary vascular architecture.^34^

### Isolation and functional analysis of cardiac mitochondria

For evaluating damage to oxidative phosphorylation (OXPHOS) machinery, quantitative analyses of complete *e.t.c.* Complexes’ activities were performed. Briefly, mitochondria were isolated from HVCM cells (Mitochondria Isolation Kit, 89879, Thermo Fisher, Waltham, MA, USA) and subjected to analysis of Complex I (AAMT001, Millipore), Complex II and III (ab109905, Abcam), Complex IV (ab109911, Abcam) and Complex V (AAMT005, Millipore) activities using commercially available kits.

For validating *in vitro* findings, mitochondria were isolated from right ventricles (Mitochondria Isolation Kit, 89801, Thermo Fisher) and were subjected to quantitative evaluation for changes in mitochondrial *e.t.c.* Complexes by western blot (Total OxPhos Complex Kit, 458099, Invitrogen, Waltham, MA, USA). Analysis of Complex I–V activities was performed as mentioned above. Further, changes in gene and protein expression levels of key regulators of mitochondrial bio-genesis (mtTFA, Nrf1, Nrf2 and PGC1α), fatty acid metabolism (PPARα/β/γ), redox-function (Nox-2, Cox-2), bio-energetic function (UCP-2, UCP-3) and cellular proliferation (Bcl2/Bax) were investigated via polymerase chain reaction (PCR) and western blot, respectively.

### Semi-quantitative polymerase chain reaction

A detailed description can be found in the Supplementary Material and list of primers can be found in Supplementary Table T1.

### Immunoblot analysis

A detailed description can be found in the Supplementary Material.

### Statistical analysis

Data are expressed as the means±standard deviations (s.d.’s) for each experimental group performed in triplicate. The results were analyzed for statistical significance using one-way ANOVA. Values were considered to be statistically significant at *P⩽0.05 vs NVC, **P⩽0.01 vs NVC, ^#^*P*⩽0.05 vs HVC and ^##^*P*⩽0.01 vs HVC. Non-significant changes are depicted as NS.

## Results

### Characterization of NCF

The FTIR spectrum of NCF was expected to elucidate the complex appearance of variable peaks as characteristics of PQQ (containing multiple bonds including N-H and C-N groups) and signature peaks of curcumin.

**Figure 1:**
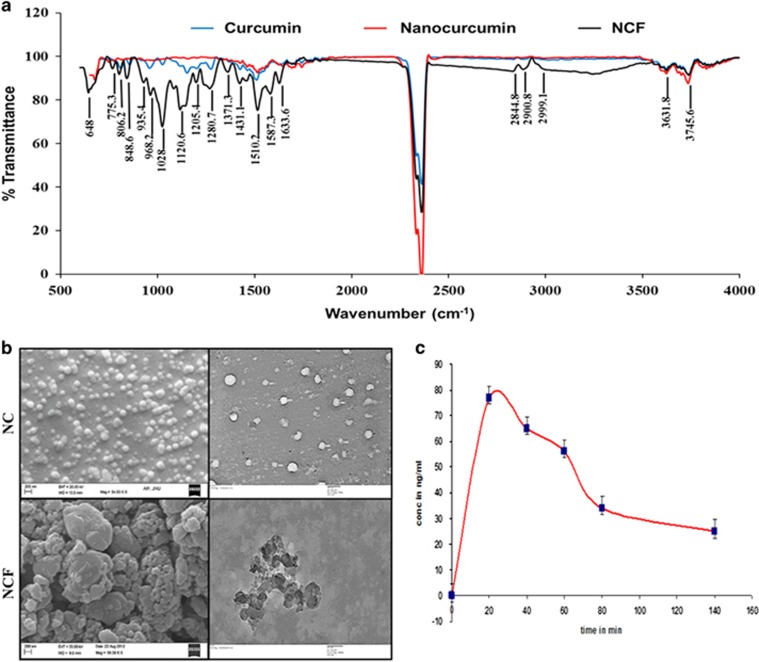
The FTIR analysis of nanocurcumin formulation (NCF) (**a**) along with SEM and TEM (**b**) and the plasma retention kinetics of NCF in rats (**c**).

The FTIR spectrum of NCF revealed distinct patterns of broad and sharp peaks ranging from 650 to 4000 cm^−1^. The appearance of distinctly sharp peaks at 648 and 1028 cm^−1^ depicted the presence of C-H bends and C-N stretches, respectively.^35^ A strong peak at 1280.7 cm^−1^ indicated a C-H wag structure.^36^ The consecutive appearance of strong peaks at 1371.3 and 1431.1 cm^−1^ indicated stretching vibrations of C-H_3_ and C-C bonds, respectively. The appearance of broad peaks at 1633.6 cm^−1^ and a strong peak at 1587 cm^−1^ depicted stretching vibrations of C-C and N-H bend structures.^35^ A broad peak ranging from 2800 to 3000 cm^−1^ depicted the presence of H-C=O:C-H bends along with C-H stretches. Peaks in the range of 3500–3750 cm^−1^ appeared as characteristic for curcumin, with strong peaks at 3631 and 3745 cm^−1^, indicating vibrations of O-H stretches in ring structures^35^ (Figure 1a).

The DLS analysis consistently depicted improvements in physico-chemical properties of NCF in terms of zeta potential, particle size, electrophoretic mobility and conductivity compared with NC. The zeta potential of NCF was −50.3 mV (−30 mV in NC), with an average particle size 1955 nm (212 nm in NC). The electrophoretic mobility and conductivity of NCF were −3.941 μm cm V^−1^ s^−1^ (−2.348 μm cm V^−1^ s^−1^ in NC) and 0.404 mS cm^−1^ (0.0359 mS cm^−1^ in NC), respectively (Table 1). Similarly, homogenous populations of NCF particles were visible in SEM and TEM images (Figure 1b). The DLS analysis certainly depicted negative zeta potential with efficient electrophoretic mobility and conductivity, which represent evidence of high NCF bio-stability. This finding was further corroborated with a pharmacokinetic assessment (Figure 1c) of NCF, indicating a high mean resident time, clearly suggesting improved NCF bio-availability and bio-stability (Table 2).

### Toxicity analysis

NCF was physiologically safe and did not cause any toxicity in HVCM cells. HVCM cells did not exhibit any evidence of micro-nuclei or DNA damage and maintained normal cellular division (Supplementary Figure S1). No significant changes in LDH activity or cellular viability were observed in HVCM cells (Supplementary Figure S1). Similarly, the physical appearances, body weight and behavioral tendencies and the blood gas contents and hematological parameters were normal in NCF-supplemented animals compared with control animals (Supplementary Tables T2–T4). No clinically relevant damage to vital organs (heart, lungs and liver) was observed in NCF-supplemented animals compared with normal animals (Supplementary Figure S2).

### NCF imparts cyto-protection under hypoxic conditions

Hypoxia stress decreased HVCM cellular viability (decreased to 20 % compared with NVC). Hypoxia-mediated cell death was confirmed using caspase-3, -7 activation by FACS and was further confirmed by TUNEL assay. Significant NCF uptake was observed in HVCM cells (Supplementary Figure S3) under hypoxic conditions. NCF treatment (500 ng ml^−1^) significantly improved cellular viability (97%) compared with NC- and PQQ-treated cells under hypoxic conditions compared with HVC (76% and 69%, respectively). Corroborating these findings, decreased caspase-3, -7 activation and TUNEL positivity in NCF-treated cells further confirmed the enhancement in cellular viability under hypoxic conditions compared with NC- and PQQ-treated cells (Supplementary Figure S3).

### NCF protects from hypoxia-induced hypertrophy

**Figure 2:**
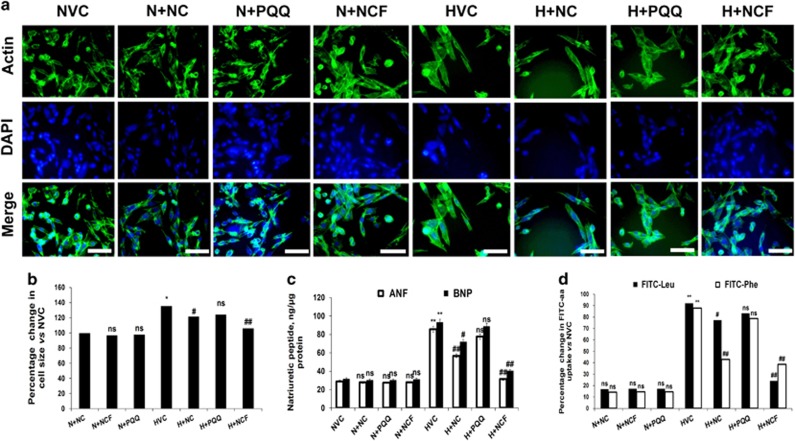
The effect of nanocurcumin formulation (NCF) on hypoxia-induced hypertrophy in human ventricular cardiomyocytes (HVCM) cells. Increments in cell size (× 40) (**a**, **b**) along with atrial natriuretic factor (ANF) and brain natriuretic peptide (BNP) levels (**c**) depicted hypertrophic growth. Increments in FITC-leucine and FITC-phenylalanine uptake further confirmed hypoxia-induced hypertrophy (**d**). NCF supplementation effectively modulated hypoxia-induced hypertrophy *in vitro*. Data are expressed as the means±s.d. Values were considered to be statistically significant at *P⩽0.05 vs NVC, **P⩽0.01 vs NVC, ^#^*P*⩽0.05 vs HVC and ^##^*P*⩽0.01 vs HVC. Non-significant changes are depicted as NS. Scale bar, 20 μm.

Induction of hypertrophy was evident in HVCM cells under hypoxic conditions, as indicated by morphometric analysis (by 35%) and the increases in FITC-leucine (by 63%) and phenylalanine (by 65%) uptake and was confirmed from the increases in ANF (by 85%) and BNP (by 93%) levels compared with the NVC group (Figure 2a–d). Significant declines in cell size (by 30%) and FITC-leucine (by 23%) and phenylalanine (by 24%) uptake predicted decreased hypertrophy in NCF-supplemented cells under hypoxic conditions compared with the HVC group (Figure 2a–d). Importantly, PQQ treatment effectively improved cellular viability but failed to demonstrate anti-hypertrophic effects.

**Figure 3:**
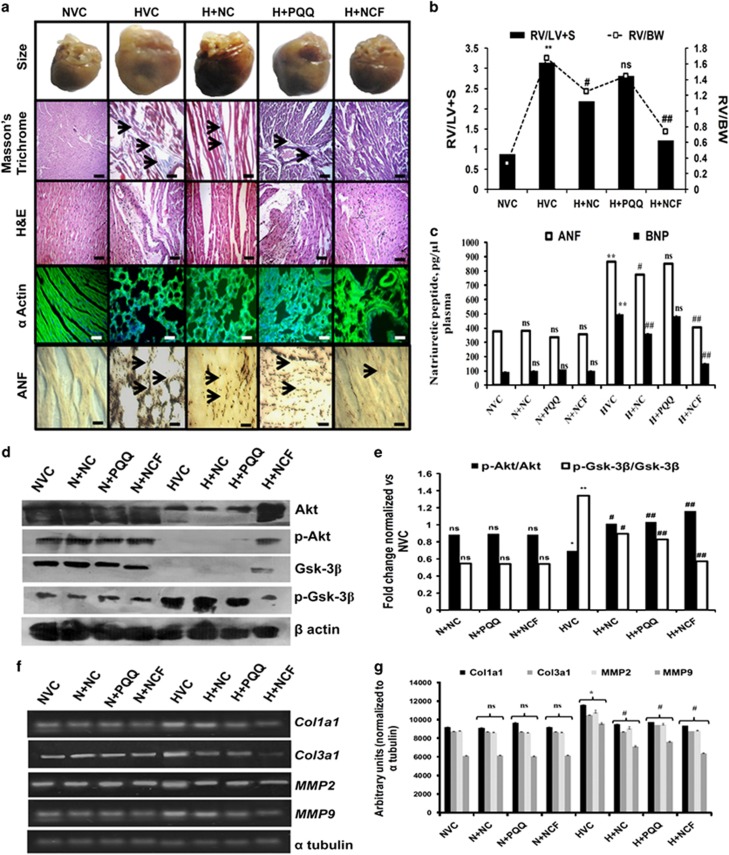
Representative figures showing the effect of nanocurcumin formulation (NCF) supplementation on cHH-induced RVH in rats. Chronic HH-mediated RVH was evident in animals by increases in heart size, collagen accumulation (arrows), Fulton’s index and histopathology staining (× 40) (**a**, **b**). Tissue expression levels of skeletal α-actin and atrial natriuretic factor (ANF) were increased due to hypertrophic growth (**b**). Circulating levels of ANF and brain natriuretic peptide (BNP) further confirmed cHH-induced RVH in rats (**c**). Phosphorylation and activation of Akt-Gsk signaling promoted hypertrophic growth in rats under hypoxic conditions (**d**, **e**). Changes in gene expression levels of markers of myocardial matrix remodeling (*Col1a1* and *Col3a1*, along with *MMP2* and *9*) (**f**, **g**) in various experimental groups. Data are expressed as the means±s.d. NCF supplementation modulated chronic hypobaric hypoxia (cHH)-induced right ventricular hypertrophy (RVH) better than nanocurcumin (NC) and pyrroloquinoline quinone (PQQ) treatments. Values were considered to be statistically significant at **P⩽0.01 vs NVC, ^#^*P*⩽0.05 vs HVC and ^#^*P*⩽0.01 vs HVC. Non-significant changes are depicted as NS. Scale bar, 10 μm.

Similarly, cHH-induced RVH was evident in animals due to the more than four-fold increases in RV/LV+S and RV/BW content (Figure 3b). Simultaneous increase collagen accumulation of both types and tissue ANF and α-actin expression levels confirmed the hypertrophic growth, along with the increased gene expression levels of myocardial markers of matrix remodeling, that is, collagen type 1 (*Col1a1* and *Col3a1)* (up to 1.8-fold) and 3 with matrix metallopeptidase (MMP) 2 and 9 (up to 3.3-fold) compared with normoxia control animals (Figure 3c–g). Increases in the circulating levels of ANF (2.3-fold) and BNP (5.3-fold) confirmed cHH-induced RVH in animals (Figure 3a and c). Treatment of animals with NCF effectively reduced RV/LV+S (1.2-fold) and RV/BW (0.7-fold) contents, with parallel decreases in collagen accumulation, ANF expression and α-actin levels. These findings were validated by further significant decreases in circulating levels of ANF (1.17-fold) and BNP (1.63-fold) and the expression levels of *Col1a1* and *Col3a1* (up to 1.6-fold) and MMPs (up to 3.1-fold) in NCF-supplemented animals compared with vehicle-treated animals under cHH.

The improvement in NCF efficacy was further corroborated based on the Akt/Gsk-3β-signaling pathway. cHH promoted p-Gsk-3β/Gsk-3β upregulation and downregulated p-Akt/Akt contents (Figure 3d and e). NCF supplementation showed significant restoration in the p-Akt/Akt and p-Gsk-3β/Gsk-3β contents (*P*⩽0.05) compared with NC-treated cells under hypoxic conditions, clearly demonstrating the enhanced cardio-protective efficacy of NCF under cHH.

### cHH-induced RVH in animals due to increased pulmonary resistance

**Figure 4:**
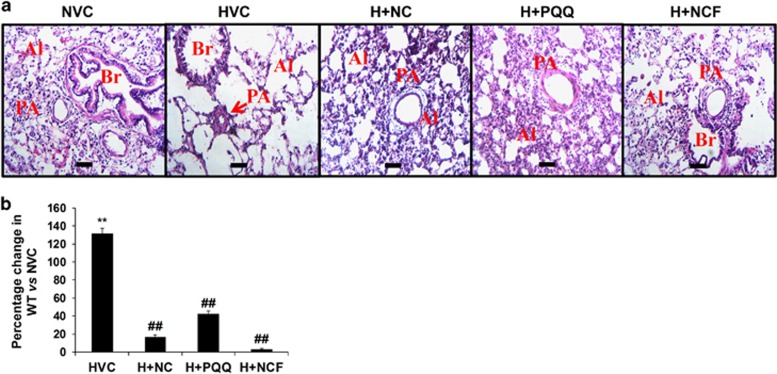
Figure showing changes in lung histology in various experimental groups (**a**), as evident by morphological changes in alveolar spaces (Al, red) and bronchioles (Br, red). Histopathological staining (× 40) revealed increases in pulmonary arteries (PA, red) and medial wall thickness (WT) in HVC groups, while nanocurcumin formulation (NCF) supplementation showed significant decreases compared with HVC, NC and PQQ (**b**). Data are expressed as the means±s.d. Values were considered to be statistically significant at **P⩽0.01 vs NVC and ^##^*P*⩽0.01 vs HVC. Scale bar: 10 μm.

The pulmonary vasculature underwent discrete morphometric and architectural changes under cHH. H&E staining in rat lungs revealed vascular remodeling of the pulmonary bed. The alveoli were scattered in appearance, fewer in number and larger in size (Figure 4a). The pulmonary arterial lumen appeared narrower, and the pulmonary artery (PA) medial wall underwent muscularization, thus promoting resistance to blood flow. Exposure to cHH led an increase in PA medial wall thickness (WT), which increased by 132% (*P*⩽0.01) in experimental animals compared with NVC (Figure 4). Importantly, animals supplemented with NCF showed a sharp decline in WT (2.8%) (*P*⩽0.01) and maintenance of the vascular architecture when compared with HVC (2.8%) (Figure 4a and b). The WT declined to 16% and 42% in NC- and PQQ-supplemented animals, respectively (*P*⩽0.01), when compared with HVC controls. These qualitative and quantitative data suggest that pulmonary vascular resistance increased under cHH due to narrowing of the PA. The results herein provide evidence that NCF supplementation effectively induced morphometric and functional modulation in pulmonary vasculature animals under cHH better than NC and PQQ alone.

### NCF protects from hypoxia-induced stress by maintaining mitochondrial function

**Figure 5:**
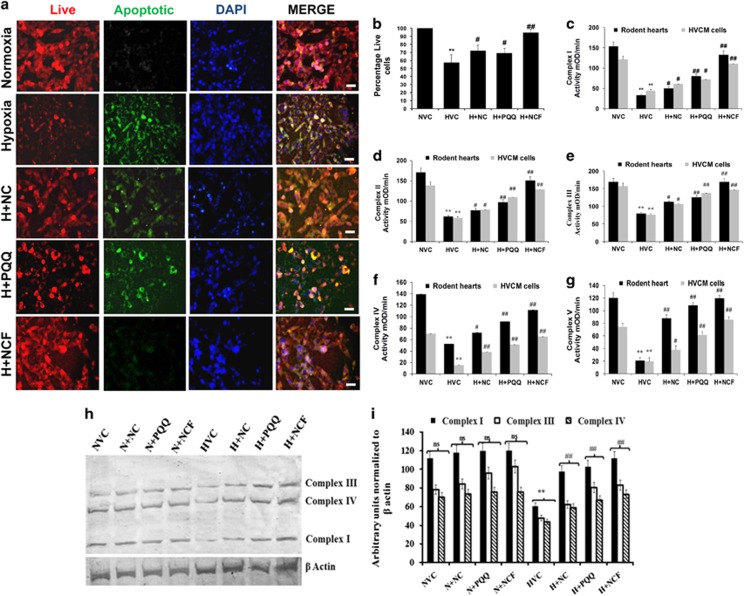
Figure showing changes in mitochondrial homeostasis under hypoxic conditions. Human ventricular cardiomyocytes (HVCM) cells experienced apoptotic cell death due to hypoxia-mediated impairments in membrane potential (× 40) (**a**, **b**) depicting damage to mitochondrial homeostasis. Hypoxia-induced modulations in mitochondrial electron transport chain (*e.t.c.*) Complexes’ activities (**c**–**g**) were also evident both *in vitro* and *in vivo.* (**h**, **i**) The effects of cHH-induced modulation on regulators of oxidative phosphorylation. Data are expressed as the means±s.d. Values were considered to be statistically significant at **P⩽0.01 vs NVC, ^#^*P*⩽0.05 vs HVC and ^##^*P*⩽0.01 vs HVC. Non-significant changes are depicted as NS. Scale bar, 10 μm.

**Figure 6:**
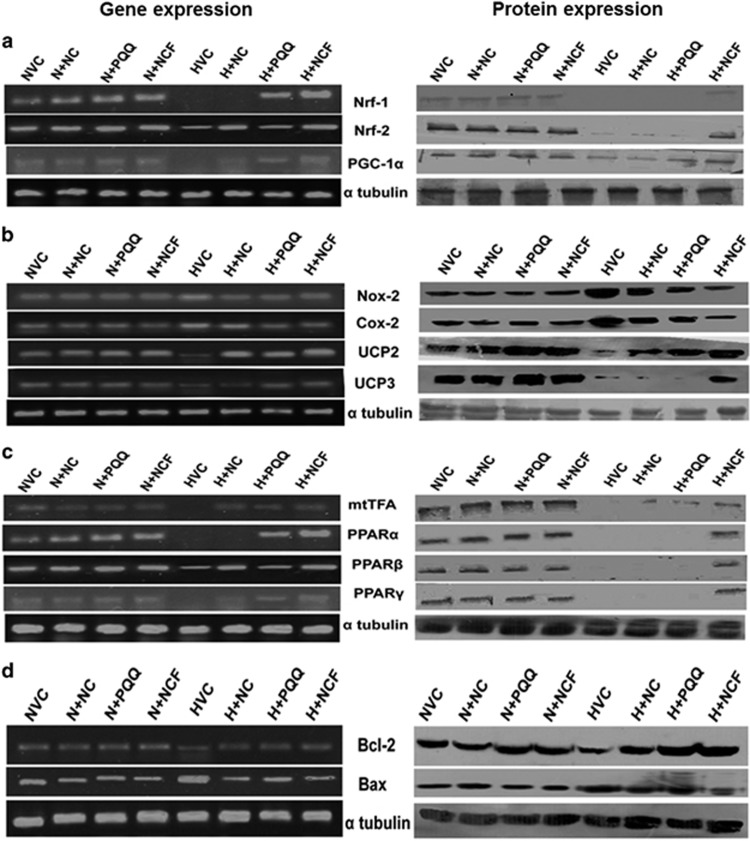
Figure showing gene and protein expression changes in mitochondrial homeostasis regulators: chronic hypobaric hypoxia (cHH)-mediated decreases in regulators of mitochondrial biogenesis (mtTFA, Nrf1, Nrf2 and PGC1α) (**a**), redox function (Nox-2 and Cox-2) (**b**), fatty acid regulation (PPARα/β/γ) (**c**) and cell survival (Bcl2, Bax) (**d**) were evident in both gene and protein expression studies. Values were considered to be statistically significant at **P⩽0.01 vs NVC, ^#^*P*⩽0.05 vs HVC and ^##^*P*⩽0.01 vs HVC. Non-significant changes are depicted as NS.

Hypoxic stress promoted damage to HVCM cells by disrupting the mitochondrial membrane potential (ψ_m_), thus promoting mitochondrial damage-mediated apoptosis (Figure 5a and b). Excessive ROS leakage and decreased MnSOD activity further validated hypoxia-mediated mitochondrial damage to HVCM cells (Supplementary Figure S4). Further, exposure of HVCM cells and rodent hearts to cHH resulted in decreased activities of Complex I–V, depicting damage to mitochondrial chief functional units (Figure 5c–i). The data are in accordance with previously described damage to *e.t.c.* Complexes in rodent skeletal muscles.^37^ Accompanying these findings, we found that Nox-2 and Cox-2 expression levels increased under cHH, suggesting compromised functions of Complexes I and IV, respectively (Figure 6a–d and Supplementary Figure S5A–H), along with decreased expression levels of *e.t.c.* Complexes in rodent hearts (Figure 6e and f). Decreased levels of PPARα/β/γ, mtTFA, Nrf1, Nrf2 and PGC1α clearly depict impaired mitochondrial biogenesis, while decreased levels of UCP-2 and UCP-3 clearly demonstrate diminished bio-energetic efficacy due to the impaired function of Complex V under hypoxic conditions.

NCF treatment significantly restored *e.t.c.* Complexes’ activity levels both *in vitro* and *in vivo* better than NC or PQQ alone (Figure 5c–g). Better regulation in ψ_m_ was evident in NCF-treated HVCM cells under hypoxic conditions compared with NC or PQQ or vehicle (Figure 5a and b). Importantly, the markers of mitochondrial bio-genesis and functional control (PPARα/β/γ, mtTFA, Nrf1, Nrf2 and PGC1α), along with bio-energetic efficiency (UCP-2,-3) and cell-survival (Bcl2, Bax), were more efficiently restored in NCF-treated animals than in those treated with NC and PQQ alone compared with vehicle under cHH (Figure 6a–d and Supplementary Figure S5A–H). NCF-supplemented animals exhibited restoration of the expression levels of Complexes I–V along with their activities better than either treatment alone (Figure 5h and i). Collectively, these data demonstrate robust improvements in mitochondrial protective efficacy of NCF in cardiomyocytes compared with NC and PQQ.

### NCF supplementation prevents NFκB-p65 activation under hypoxic conditions

**Figure 7:**
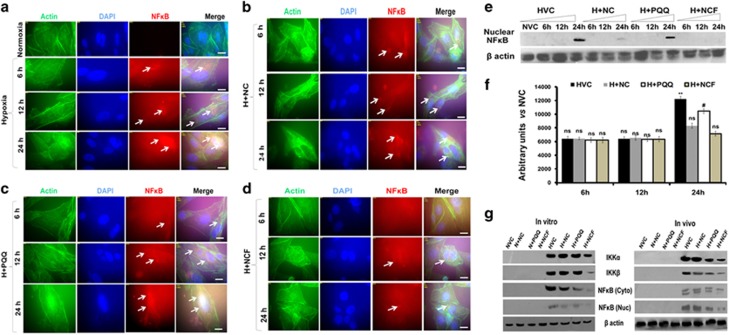
Representative figure showing NFĸB activation under hypoxic conditions: human ventricular cardiomyocytes (HVCM) cells experienced cytoplasmic NFĸB activation as early as 6 h following the onset of hypoxia (× 100) (**a**–**d**), while nuclear translocation was observed only at 24 h following onset (**e**, **f**). Rodent right ventricles and HVCM cells demonstrated NFĸB activation under chronic HH or hypoxic exposure for 24 h, respectively (**g**). Supplementation of HVCM cells and rats with nanocurcumin formulation (NCF) resulted in markedly decreased NFĸB activation, followed by NC and PQQ alone. Scale bar, 20 μm.

The cellular activation and cytoplasmic accumulation of NFκB-p65 activation and cytoplasmic accumulation started as early as 6 h following the onset of hypoxia and reached their peaks by 24 h (Figure 7a–g and Supplementary Figures S6 and S7) in HVCM cells. However, nuclear translocation of NFκB-p65 was observed only in cells exposed to hypoxia for 24 h (Figure 7a–g).

**Figure 8:**
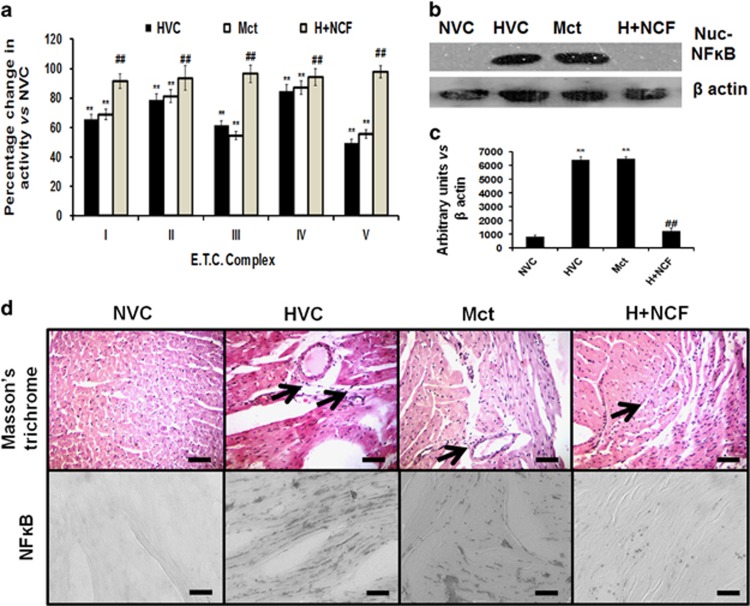
The comparative pathogenicity in chronic HH- and monocrotaline-induced RVH models: Chronic HH- and monocrotaline (Mct)-induced RVH promoted increases in mitochondrial electron transport chain (*e.t.c.*). Complexes’ I–V activity levels (**a**). Both models showed NFĸB nuclear accumulation and tissue expression, along with increased tissue collagen accumulation (× 40) (arrows) (**b**–**d**). Data are expressed as the means±s.d. Values were considered to be statistically significant at **P⩽0.01 vs NVC and ^##^*P*⩽0.01 vs HVC. Scale bar, 10 μm.

Similarly, our *in vivo* findings confirmed NFκB activation in the right ventricles under cHH (Figure 7f and g and Supplementary Figure S8). To confirm the pathogenicity of cHH-induced RVH in rats, we compared NFκB tissue expression and changes in mitochondrial *e.t.c.* Complexes’ activities along with histopathological changes in a pre-established model of monocrotaline-induced RVH.^38^ Monocrotaline infusion promoted RVH, decreased *e.t.c.* Complex activities and increased NFκB expression in the right ventricles (Figure 8a–d). Together, these data clearly depict cHH-induced RVH and damage-mediated pathogenicity in rat hearts similar to a previously established model of monocrotaline-induced RVH and failure.^39^

NCF treatment resulted in significant decreases in cytoplasmic and nuclear levels of NFκB-p65 compared with vehicle/NC/PQQ-treated cells and animals (Figure 7a–f), but NC and PQQ treatments failed. Collectively, the data demonstrate that cHH-mediated RVH and cardiomyocyte damage are dependent on NFκB-p65 activation, similar to monocrotaline-induced RVH (Figure 8b and c).

## Discussion

Cardiac hypertrophy appears as an initial adaptive response to hypoxia, but prolonged stress induces de-compensation and irreversible damage. The increase in systemic oxygen demand under hypoxic stress is well elucidated.^24^ To meet this increased demand for oxygenated blood, the heart frequently pumps more blood toward the lungs to improve systemic oxygenation. The heart undergoes hypertrophy under this sustained workload, with special relevance to the right ventricle.^40^ However, chronic hypoxic stress also promotes a decline in the oxygenation capacity of the lungs under chronic hypoxic stress due to excessive fluid retention, which causes an increase in the pulmonary resistance to the blood flowing from the right ventricles. However, to ensure an uninterrupted systemic blood supply, the hypertrophied right ventricle continuously pumps blood towards the lungs. Under this state of increased volume and pressure overload, the right side of the heart eventually undergoes de-compensation and suffers from pathological damage.^40, 41^ Herein, we observed an increase in the pulmonary artery medial wall thickness and a decrease in the pulmonary artery lumen, which promotes an increase in the pulmonary resistance to blood flow. These changes might have led to sustained pressure and volume overload on the right ventricle, which eventually stimulated de-compensation in the chamber.^40, 41^ Thus, the data in the present study provide evidence that the heart undergoes pathological damage due to de-compensation arising in the right ventricle that is secondary to the increased pulmonary vascular resistance. However, increased pulmonary resistance is directly associated with pulmonary hypertension; further investigations are needed to clarify the potential effects of cHH-induced hemodynamic changes on pathological RVH.^40, 41^

In the present study, we found that severe damage to Complexes I–V clearly demonstrated that hypertrophied cardiomyocytes underwent mitochondrial stress under hypoxic conditions, both *in vitro* and *in vivo*. Previous studies have shown that stress-induced decreases in the functions of these Complexes are inseparably associated with cardiomyopathies and heart failure.^2, 9, 42, 43^ However, to the best of our knowledge, this study provides the first report that chronic hypoxic stress promoted mitochondrial stress by impairing *e.t.c.* Complex activities in hypertrophied cardiomyocytes. Since optimal cardiomyocyte functioning relies on a constant supply of the optimum amount of oxygen, hypoxia-mediated oxidative stress might have contributed to such severe damage to the *e.t.c.* complex activities. The smooth flow of electrons through all five Complexes determines optimal cardiomyocyte function.^44^ As hypoxic stress promotes severe oxidative damage, excessive free-radical leakage-mediated redox imbalances might have initiated the mitochondrial damaging events that further led to impairments in the *e.t.c.* Complex activities. Further, chronic hypoxic stress impaired not only mitochondrial regulators of cell-survival, redox-maintenance and metabolism, but severe damage to regulators of mitochondrial bio-genesis was also evident. Decreased expression levels of the *e.t.c.* Complexes along with their activities clearly demonstrate that impaired mitochondrial homeostasis remains a critical molecular event behind cHH-induced de-compensatory RVH and damage.

Further molecular investigation suggested that NFκB-p65 activation emerged as a potential regulator of hypoxia-induced hypertrophy, both *in vitro* and *in vivo*. Importantly, *in vitro* activation of NFκB-p65 and stabilization of IKKα/β were observed as early as 6 h following the onset of hypoxia. These data and our previous report strongly suggest that hypoxia-mediated onset of hypertrophy and activation of NFκB-p65 in HVCM cells seems to be synchronous events (6 h).^3^ To ensure the role of NFκB-p65 activation in our animal model of RVH, we compared our findings with a previously established model of monocrotaline-induced RVH. Interestingly, monocrotaline-induced RVH was accompanied by NFκB-p65 activation and damage to *e.t.c.* Complex machinery, similar to our model of cHH-induced RVH.^45^ These findings suggest that the pathological events arising in the cHH-induced model of RVH show pre-clinical similarity to a drug-induced model of RVH. However, since monocrotaline-induced RVH remains secondary to pulmonary hypertension, it is important to assess quantitative hemodynamic changes in pulmonary arterial pressure in a cHH-induced model of RVH. Collectively, the present study shows that hypoxic stress was sufficient to induce NFκB nuclear translocation and to initiate the transcriptional activation of downstream signaling genes, thus promoting hypertrophy and damage in rats. These damages appeared in the form of de-compensated RVH along with mitochondrial damage and demonstrate the transition of cardiomyocyte hypertrophy from an adaptive physiological state to a maladaptive pathological state.

This information and synchronous molecular events suggest that chronic hypoxia-mediated de-compensatory RVH appeared in animals in response to excessive pressure and volume overload, secondary to enhanced pulmonary vascular resistance. This de-compensation mediated pathological damage in the right heart by impairing *e.t.c.* Complexes. These data suggest that although stress-induced activation of NFκB is necessary for onset of hypertrophy, chronic stress-mediated sustained NFκB upregulation might have promoted de-compensation and pathological damage.^16^ However, the exact molecular events behind NFκ-mediated pathology require further investigations.

NCF supplementation was highly effective in modulating hypoxia-induced hypertrophy compared with NC and PQQ alone, clearly depicting an improvement in the cyto-protective efficacy of NCF. Importantly, PQQ treatment showed an improvement in cellular viability under hypoxic conditions but failed to modulate hypertrophy. These findings suggest that though oxidative stress plays a crucial role in the pathogenicity of cardiac hypertrophy, other molecular events might collectively play key regulatory roles in promoting pathogenicity under hypoxic conditions. Similarly, NC supplementation showed modulations of hypertrophy and pathology but did not impart sufficient therapeutic regulation of mitochondrial health under hypoxic conditions. Together, these findings emphasize the inference that NCF supplementation could provide comprehensive therapeutic benefits under stress. NCF supplementation imparted simultaneous protection from cHH-induced damage to pulmonary architecture and damage to the right side of the heart. Robust improvements in *e.t.c.* Complex expression and activity levels with NCF treatment clearly demonstrate improvements in the therapeutic and cyto-protective potentials of NCF under hypoxic conditions compared with NC or PQQ alone. The data collectively suggest the potential efficacy of combinatorial therapeutics to combat multi-factorial pathologies mediated by hypoxic stress and further strengthen our hypothesis that combinatorial therapeutics might be an effective solution to hypoxia-mediated clinical problems.

## Publisher’s note

Springer Nature remains neutral with regard to jurisdictional claims in published maps and institutional affiliations.

## Figures and Tables

**Table 1:** Changes in zeta potential, mobility, size and conductivity of NCF compared with NC by dynamic light scattering

*Material*	*Zeta potential (mV) (mean±s.d.)*	*Mobility (μm cm V*^*−1*^* s*^*−1*^*) (mean±s.d.)*	*Size (nm±s.d.)*	*Conductivity (mS cm*^*−1*^*) (mean±s.d.)*
NC	−30±2.33	−2.348±0.24	212±221	0.0359±0.0002
NCF	−50.3±2.41	−3.941±0.36	1955±238	0.404±0.001

Abbreviations: NC, nanocurcumin; NCF, nanocurcumin formulation.

Data are represented as the means±s.d. from three independent experiments.

**Table 2:** Qualitative data representing the pharmacokinetic profile of NCF

*Parameters*	*Results*
*k*_el_	0.017±0.007
AUC_0–*t*_ (mg ml^−1^ min^−1^)	1.19±0.44
AUC_0–∞_ (mg ml^−1^ min^−1^)	2.55±0.63
*t*_1/2_ (min)	28.55±18.11
*V*_d_ (l kg^−1^)	40.11±13.29
*C*_l_ (l kg^−1^ min^−1^)	84±27.27
MRT (min)	58.22±29.56
*k*_α_ (l min^−1^)	0.22±0.10
*C*_max_ (mg ml^−1^)	0.03±0.002
*t*_max_ (min)	13.09±4.1

Abbreviations: AUC, area under curve; MRT, mean residence time; NC, nanocurcumin; NCF, nanocurcumin formulation.

Data are represented as the means±s.d. from three independent experiments.
