# Supplementary material for: Nanocurcumin–pyrroloquinoline formulation prevents hypertrophy–induced pathological damage by relieving mitochondrial stress in cardiomyocytes under hypoxic conditions
Source: Exp Mol Med. 2017 Dec 1;49(12):e404–. doi: 10.1038/emm.2017.199 (PMC5750470; doi:10.1038/emm.2017.199)
Supplement: Supplementary Information [file emm2017199x1.docx]

**Methods**

In order to prepare NCF, 30 mg of PQQ was added to a suspension of NC in ultrapure de-ionized double distilled water (30 %, w/v) under vigorous homogenization at 14,000 rpm, 4 °C for 30-40 minutes (RQ-140/D-FP, REMI Instruments, India) under sterile conditions. The suspension was aseptically lyophilized at -25 °C and the powdered NCF formulation so obtained was stored in ultra-clean, de-contaminated vacuum desiccator in dark.

In order to confirm binding of NC to PQQ, FTIR-spectrum of NCF was recorded on IR spectrophotometer (Bruker 250, Germany) with a scanning range of 400-5000 cm^-1^ and 128 scans/recording. Dynamic light scattering (DLS) analysis was performed to check the zeta potential, size and electrophoretic mobility of NCF with Zetasizer Nano ZS (Malvern Instruments Ltd., UK). Morphological appearance of NCF was analysed by scanning electron microscopy (SEM) (S-360, Cambridge) and tunnelling electron microscopy (TEM) as previously described^1^.

***In vitro* uptake and toxicity analysis of NCF**

Cyto-toxicity of NCF in HVCM cells was estimated using neutral red uptake assay and lactate dehydrogenase activity assay (MAK066, Sigma Aldrich) at doses of 100, 500 and 1000 and 5000 µM for 24 and 48 h^2^. Genotoxicity assessment was performed using *in vitro* micronucleus (IVMN) assay in cells treated with 5000 µM of NCF for 72 h with simultaneous assessment of cellular division following Organization for Economic Co-operation and Development (OECD) guidelines. Briefly, mono-nucleated and metabolically active HVCM cells were grown in 6-well culture slides, allowed to reach 60-70% confluence and treated with 5000 µM of NCF for 72h without cytokinesis blocker in order to avoid chemical induced nuclear extrusion or DNA damage ^3,4^. After 72 h, the media was removed and the slides were washed twice with sterile PBS and nuclei were stained with DAPI. The slides were dried and immediately mounted in ProLong® Gold Antifade Reagent (P36934, Molecular Probes) and carefully visualized under fluorescent microscope (60X)(Motic AE31). Photographs were taken for at-least 15 different frames for same experimental group. In order to ensure normal mitotic division for validating IVMN assay, the cells were monitored for cell division and proliferation by neutral red uptake assay. Simultaneously, cells treated with NCF for 72 h were stained with apoptosis indicative dye propidium iodide (0.1%, w/v) and counterstained with DAPI and analysed by FACS (Fluorescence activated cell sorting)(FACSCalibur™, Becton Dickinson).

**Cell culture, hypoxia exposure and protein isolation**

HVCM cells were maintained under the conditions of normoxia or hypoxia as previously stated ^5^. The cells were divided into eight experimental groups, i.e preparation of nuclear and cytoplasmic protein extracts and protein estimation was done according to method described previously ^6^.

**Cellular viability and hypertrophy**

*In vitro* model of hypoxia induced hypertrophy and mitochondria mediated damage was established as previously described ^5^. Briefly, HVCM cells underwent hypertrophy and mitochondrial damage when exposed to hypoxia (0.5% O_2_ for 24 h). For dose optimization, cellular viability was assessed in NCF treated HVCM cells (100-1000 ng/ml) as compared to NC and PQQ using neutral red uptake assay and confirmed by caspase-3,-7 activation assay (APT403, Calbiochem, Millipore) by FACS and TUNEL (TUNEL-Apoptosis detection kit, 17-141, Millipore, USA). Assessment of hypoxia induced hypertrophy was done by estimation of cellular levels of ANF (Atrial natriuretic factor)(EIA-ANP-1, RayBiotech) and BNP (B-type natriuretic peptide)(EHNPPB, Pierce) using commercially available ELISA kits and further confirmed by FITC-labelled leucine (NPN-1, Millipore) and phenylalanine (NPN-2, Millipore) uptake assay as previously described ^5,6^.

**Immunocytofluorescence**

Hypoxia induced activation and nuclear translocation of NFκB-p65 was assessed by immunofluorescence in HVCM cells using mouse monoclonal anti-NFκB antibody (1:100, sc8008, Santa Cruz Biotechnology) as previously described. Images were acquired under high resolution fluorescent microscope (100X)(IMAGER.M2, AxioCam MRc5, Carl Zeiss)^5^ and further confirmed by western blot.

**Pharmacokinetic assessment of NCF in Sprague-Dawley rats**

The plasma retention of NCF was evaluated using high-performance liquid chromatography (HPLC) (Agilent, Infiently, 1200 USA) equipped with PDA-420 nm detector and reversed phase C-18 analytical column (4.6 × 250 mm, particle size 5 µm). Mobile phase consisting of 10 mM ammonium acetate pH 4.5±0.5 and acetonitrile (60:40, v/v) was used at a flow rate of 1.5 mL/min. NCF was suspended in sterile PBS and the suspension was stabilized by sonication as previously described ^5^. Animals were given single dose of NCF i.e. 100 mg/kg b.w. by oral gavage. Blood was withdrawn at time intervals of 0, 20, 40, 60, 80 and 140 min in K_2_-EDTA coated vacutainers and clear plasma was isolated by centrifugation at 500 × g for 15 minutes.

Curcumin and curcumin metabolites were extracted from plasma samples by solid phase extraction. Briefly, plasma was loaded onto a HLB cartridge (Sigma), washed with 1 ml of 40:40:20::methanol:water:glacial acetic acid and eluted with 1 ml of methanol and mixed with 1.5 % glacial acetic acid. Eluent sample was evaporated to dry at low temperature and re-suspended in 50 μl of water: acetonitrile (50:50). The samples were then centrifuged at 8000 x g for 10 min at 4˚C and the supernatant was kept for −20˚C and subjected to HPLC analysis. Standard solutions of curcumin (5-1000 ng/ml) were prepared in 1 ml rat plasma and extracted as described above.

The NCF plasma kinetics (PK_a_) was graphically plotted by fitting exponential values of plasma concentration *vs* time. The graphical method was adopted for calculating elimination half-life (t_1/2_) and residues method was adopted for the measurement of half-life of absorption, respectively. The absorption constants (k_a_) and elimination constant (*k*_el_) were calculated from the equation 0.693/t_1/2_, where 0.693 is considered as first order elimination rate constant and t_1/2_ is the half-life of absorption or elimination respectively. The simple trapezoidal rule is used for the determination of bio-availability or area under the curve (AUC) from the plasma concentration *vs* time of zero to the last sampling time (AUC_0-t_). The area under the curve from time zero extrapolated to infinity (AUC_0-∞_) was calculated by the equation

AUC_0-∞_ = AUC_0-t_

and total clearance (Cl) was calculated according to equation

dose/AUC_0-∞_

Distribution volume (V_d_) was calculated using the equation

Cl/AUC

The mean residence time (MRT) calculated from the equation

AUMC/AUC,

where AUMC is area under the first moment curve. AUC The maximum plasma concentration (C_max_) calculated from the experimental data as well as the time.

**Simulation to cHH and tissue harvesting**

The animals were initially allowed to acclimatize to HH (pO_2_ ~11.7 %) at simulated ambient pressure equivalent to 15,000 feet, at 28 ± 1° C, relative humidity 55 ± 2 % for 24 h in animal decompression chamber (Ballice Instruments, India) before exposure to cHH. Then after the animals were exposed to extreme high altitude hypoxia at simulated ambient pressure equivalent to 25,000 feet altitude (pO_2_ ~ 8%, temperature 28 ± 1° C, relative humidity 55 ± 2 %) for 3 weeks or otherwise kept in animal house and treated as normoxia ^7^. Animals were given 10 mg/kg b.w./day of NC or PQQ or NCF or vehicle. The animal decompression chamber was opened at every 24 h for 10 minutes at fixed time (10:00 am) for dosing, changing of beddings and replenishing the food and water. The pathogenicity of cHH induced RVH was compared to previously established monocrotaline-induced RVH ^8^.

Immediately after completion of exposure to cHH, the animals were sacrificed to excise lung and heart tissues, washed with ice-cold PBS and blot-dried. Blood was collected by left-ventricular puncture in heparinized tubes and plasma was isolated. Major arteries and blood were removed and hearts were separated into three parts, i.e. right ventricles (RV), left ventricle+septum (LV+S) and atria (At), snap-frozen in liquid nitrogen and stored at ­-80° C till further use.

**Semi-quantitative polymerase chain reaction**

Changes in gene expression were analysed by semi-quantitative PCR amplification of genes related to mitochondrial homeostasis, i.e. biogenesis (*PGC1α, mtTFA, Nrf1 and Nrf2*), bio-energetic deficit (*UCP-2,-3*), redox maintenance (*Cox-2, Nox-2*), fatty-acid metabolism regulators (*PPARα/β/γ*) and cell-survival (*Bax, Bcl-2*) were performed. Briefly, mRNA was isolated using Tri^®^-reagent (T9424, Sigma Aldrich). Synthesis of complementary DNA (cDNA) and PCR amplification were performed using HSRT-PCR kit (Sigma Aldrich) and performed according to manufacturer’s instructions. The list of primer sequences can be found in Supplementary table T1.

**Immunoblot analysis**

Immunoblot analysis was done in cytosolic or nuclear lysates using 30 μg proteins by resolving on 8, 10 or 12.5% SDS-PAGE (Sodium dodecyl sulphate-polyacrylamide gel electrophoresis). The nitrocellulose membranes (Millipore, USA) were probed with mouse monoclonal anti-NFκB (1:500, sc8008), mouse monoclonal anti-IKKα (1:400, sc1643), rabbit polyclonal anti-IKKβ (1:500, sc7607), goat polyclonal anti NOX-2 (1:200, sc5827), goat polyclonal anti COX-2 (1:200, sc1746), goat polyclonal anti UCP-2 (1:250, sc6525), goat polyclonal anti UCP-3 (1:250, sc31385), rabbit polyclonal anti Nrf-1 (1:200. Sc23624), goat polyclonal anti Nrf-2 (1:250, sc30915), rabbit polyclonal anti PGC-1α (1:250, sc13067), goat polyclonal anti mtTFA (1:400, sc30963), rabbit polyclonal anti PPARα (1:200, sc9000), rabbit polyclonal anti PPARβ (1:200, sc7197), rabbit polyclonal anti PPARγ (1:200, sc7196), mouse monoclonal anti-Bcl2 (1:500, K0154-3, MBL International) and mouse monoclonal anti-Bax (1:500, PA1013, MBL International) overnight at 4°C. The membranes were incubated with appropriate anti-rabbit-IgG-HRP (horse radish peroxidise)/anti-goat-IgG-HRP antibodies or anti-mouse-IgG-HRP antibodies (1:25000) at room temperature for 2 h. The membranes were washed twice and visualized by using chemiluminescent substrate (SIGMA ALDRICH, USA) and captured the image on photographic film (Kodak, USA).

**Statistical Analysis**

Data was expressed as mean ± standard deviation (SD) for each experimental group performed in triplicates. The results were analysed for statistical significance using one-way ANOVA. Values were considered to be statistically significant at ^*^p≤0.05 *vs* NVC, ^**^p≤0.01 *vs* NVC, ^#^p≤0.05 *vs* HVC and ^##^p≤0.01 *vs* HVC. Non-significant changes are depicted as ns.

**References:**

1 Akhtar F, Rizvi MMA, Kar SK. Oral delivery of curcumin bound to chitosan nanoparticles cured Plasmodium yoelii infected mice. *Biotechnol Adv* 2012; **30**: 310–320.

2 Ohno T, Futamura Y, Harihara A, Hatao M. Validation Study on Five Cytotoxicity Assays by JSAAE - Details of the Neutral Red Uptake A. *Altern Anim Test Exp* 1998; **145**: 131–145.

3 Carter SB. Effects of Cytochalasins on Mammalian Cells. *Nature* 1967; **213**: 261–264.

4 Kolber MA, Broschat KO, Landa-Gonzalez B. Cytochalasin B induces cellular DNA fragmentation. *FASEB J Off Publ Fed Am Soc Exp Biol* 1990; **4**: 3021–3027.

5 Nehra S, Bhardwaj V, Ganju L, Saraswat D. Nanocurcumin Prevents Hypoxia Induced Stress in Primary Human Ventricular Cardiomyocytes by Maintaining Mitochondrial Homeostasis. *PLoS One* 2015; **10**: e0139121.

6 Nehra S, Bhardwaj V, Kalra N, Ganju L, Bansal A, Saxena S *et al.* Nanocurcumin protects cardiomyoblasts H9c2 from hypoxia-induced hypertrophy and apoptosis by improving oxidative balance. *J Physiol Biochem* 2015; **71**: 239–51.

7 Nehra S, Bhardwaj V, Kar S, Saraswat D. Chronic Hypobaric Hypoxia Induces Right Ventricular Hypertrophy and Apoptosis in Rats: Therapeutic Potential of Nanocurcumin in Improving Adaptation. *High Alt Med Biol* 2016; **17**: 342–52.

8 Doggrell S a, Brown L. Rat models of hypertension, cardiac hypertrophy and failure. *Cardiovasc Res* 1998; **39**: 89–105.

**Legends**

**Supplementary figure S1:** Figure showing toxicity analysis of NCF in HVCM cells under hypoxia: HVCM cells were pre-treated with 5000 µM NCF for 72 h and subjected to IVMN assay (A), followed by FACS analysis for apoptosis using DAPI and PI staining (B). Optimal cell division and cellular viability were ensured by neutral red uptake assay for analyses of relative cell count at 24, 48 and 72h (C, D). Cellular toxicity was further confirmed by LDH release assay (E).

**Supplementary figure S2:** Figure showing histopathological assessment of *in vivo* toxicity analysis of NCF: Sprague-Dawley rats were supplemented with 2000 mg/kg/day NCF p.o. for 28 days and vital organs (heart, lung and liver) were analysed for structural deformities by haematoxylin and eosin staining. No sign of morphological damage were observed in NCF supplemented animals demonstrating physiological safety of NCF.

**Supplementary figure S3:** Figure showing cyto-protective and anti-hypertrophic effects of NCF: NCF supplementation improved cellular viability in HVCM cells under hypoxia (A), while high cellular uptake was observed compared to NC and PQQ (B). Decline in hypoxia induced caspase-3,-7 activation (C) and TUNEL-positive nuclei (D, E) further confirmed the improvement in cyto-protective and anti-apoptotic effects of NCF compared to NC and PQQ.

**Supplementary figure S4:** Graphical representation of changes in ROS leakage and MnSOD activity in HVCM cells under hypoxia. NCF supplementation effectively modulated ROS leakage and MnSOD activity in HVCM cells better than NC and PQQ. Data expressed as mean ± SD. Values were considered to be statistically significant at ^**^p≤0.05 *vs* NVC, ^**^p≤0.01 *vs* NVC and ^##^p≤0.01 *vs* HVC. Non-significant changes designated as ns.

**Supplementary figure S5A:** Densitometric analysis of gene expression levels of various genes responsible for mitochondrial homeostasis.

**Supplementary figure S5B:** Densitometric analysis of protein expression levels of various genes responsible for mitochondrial homeostasis.

**Supplementary figure S5C:** Densitometric analysis of gene expression levels of various genes responsible for mitochondrial homeostasis.

**Supplementary figure S5D**: Densitometric analysis of protein expression levels of various genes responsible for mitochondrial homeostasis.

**Supplementary figure S5E:** Densitometric analysis of gene expression levels of various genes responsible for mitochondrial homeostasis.

**Supplementary figure S5F**: Densitometric analysis of protein expression levels of various genes responsible for mitochondrial homeostasis.

**Supplementary figure S5G:** Densitometric analysis of gene expression levels of various genes responsible for apoptosis

**Supplementary figure S5H:** Densitometric analysis of protein expression levels of various genes responsible for apoptosis.

**Supplementary figure S6:** Figure showing qualitative assessment of *in vitro* NFĸB activation in HVCM cells under hypoxia: Hypoxic stress mediated activation of NFĸB as early as 6 h and reached peak by 24 h. NCF supplementation prevented NFĸB activation followed by NC while PQQ failed to prevent it.

**Supplementary figure S7:** Densitometric analysis of *in vitro* activation of NFĸB and its inhibitors.

**Supplementary figure S8:** Densitometric analysis of *in vivo* activation of NFĸB and its inhibitors.

**Supplementary table T1:** Table showing list of various gene primers and their accession numbers.

**Supplementary table T2**: Table showing changes in various components of blood bio-chemistry following sub-acute toxicity assessment.

**Supplementary table T3 and 4:** Table showing changes in various components of blood bio-chemistry following sub-acute toxicity assessment.

**
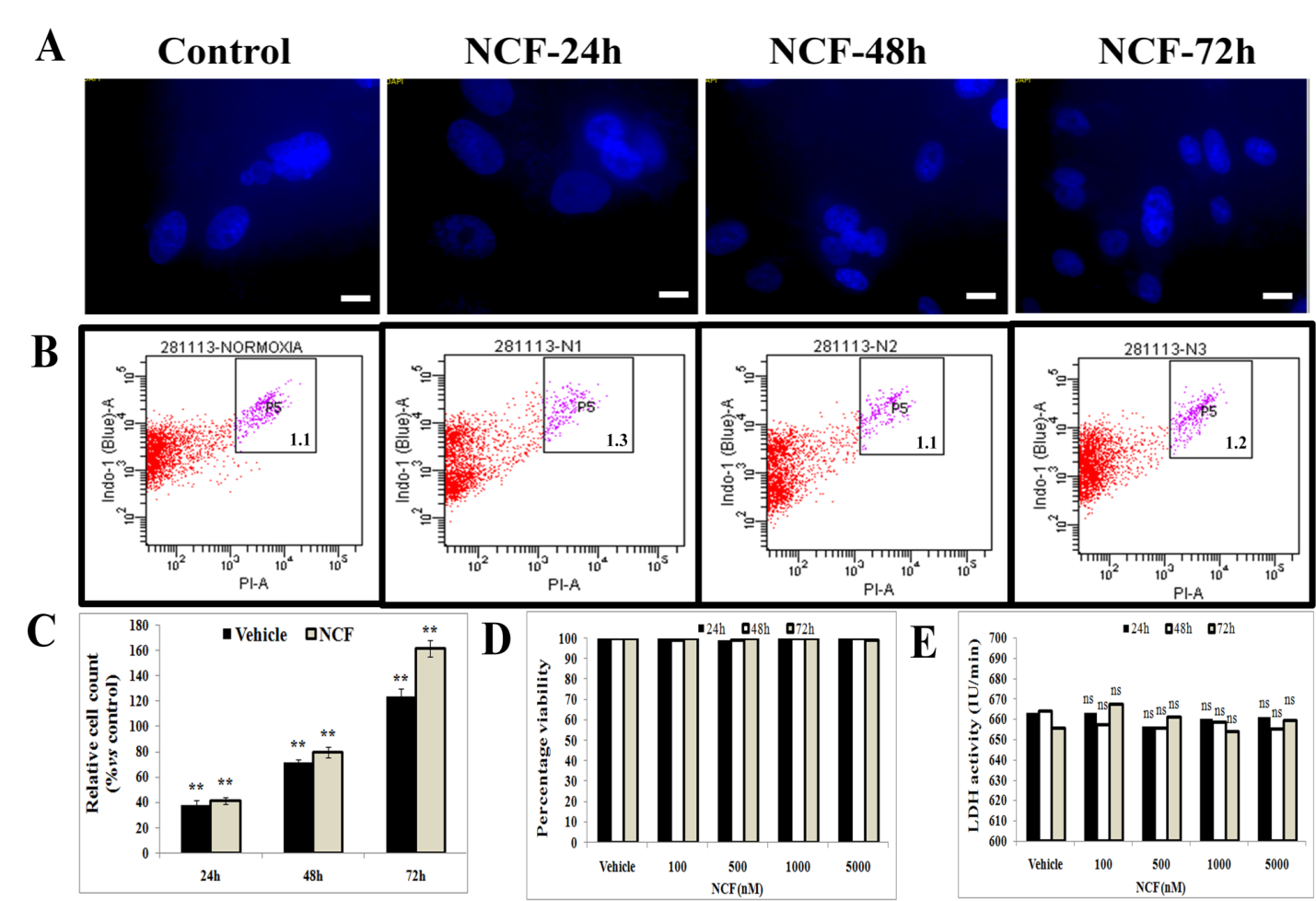
**

**Supplementary figure S1**

**
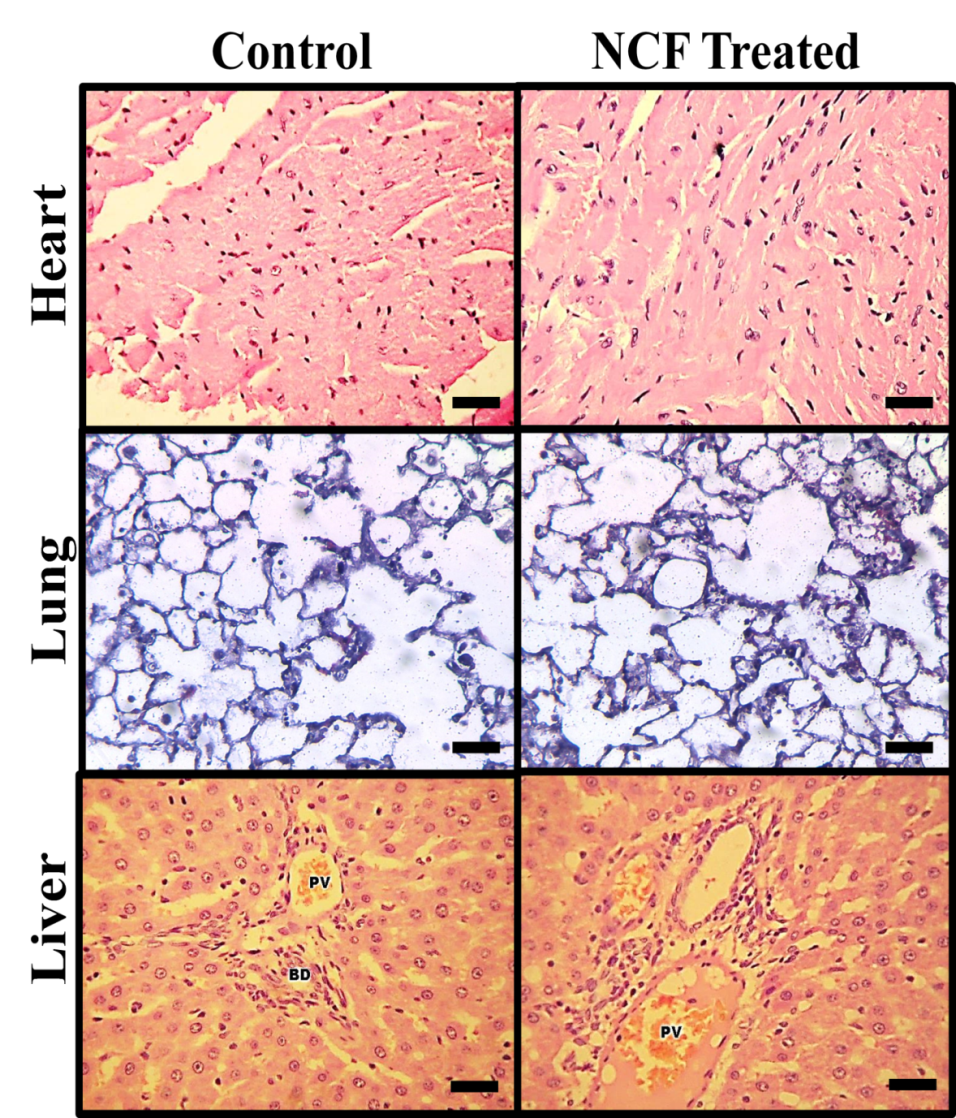
**

**Supplementary figure S2**

**
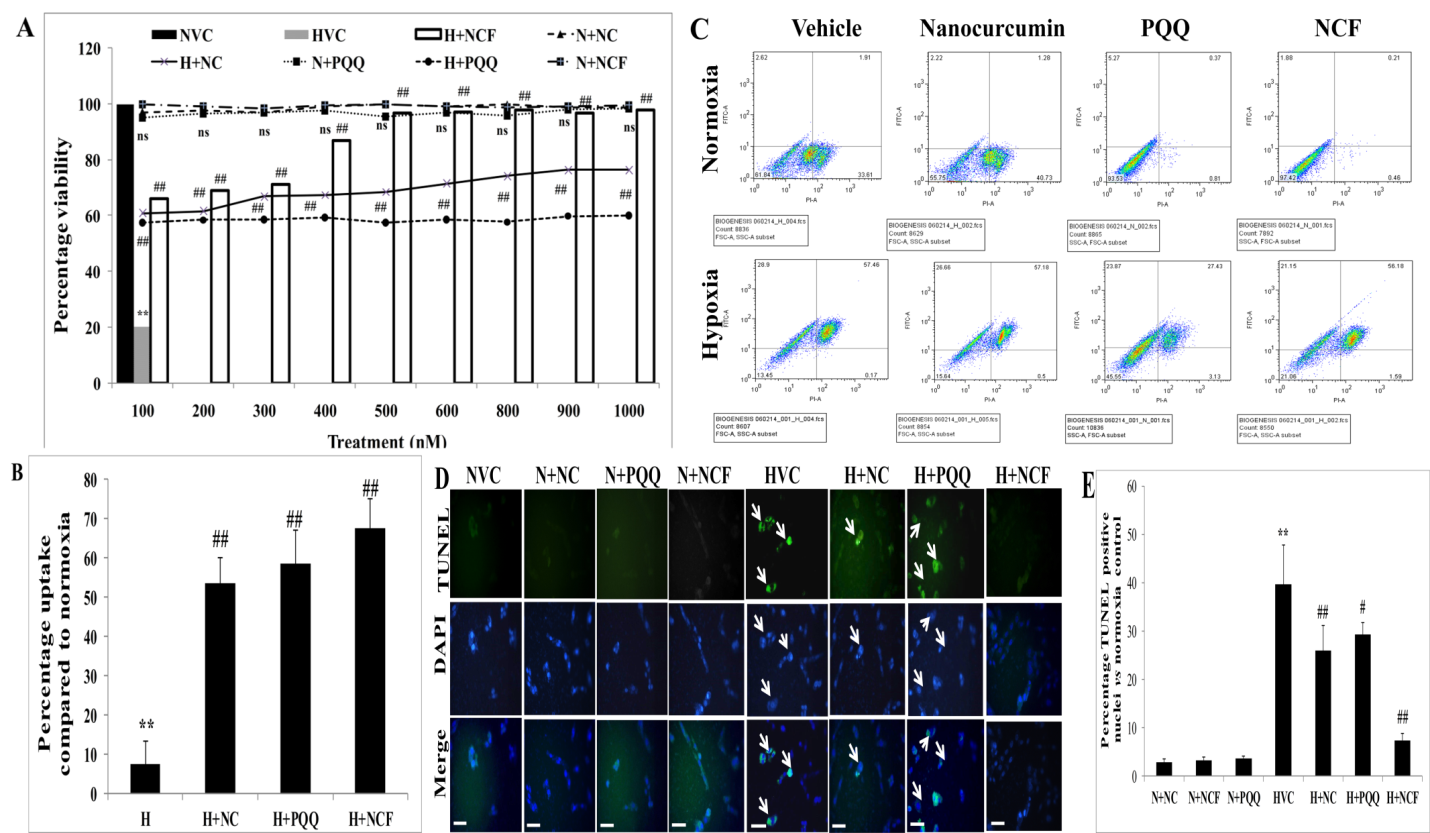
**

**Supplementary figure S3**

**
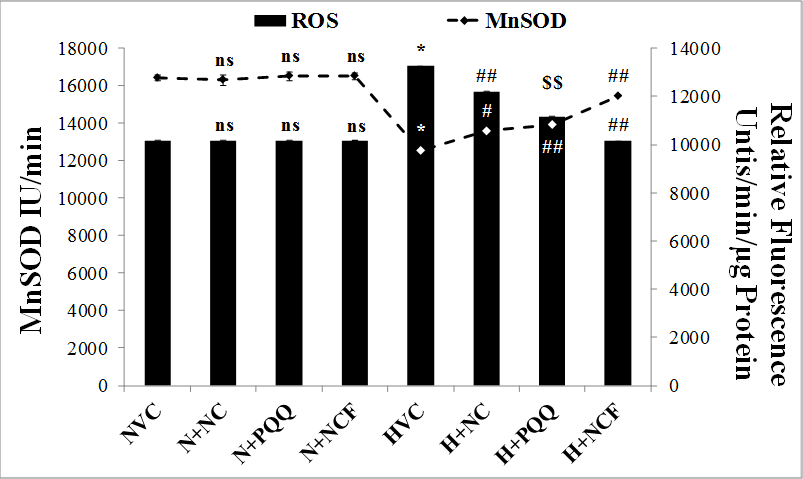
**

**Supplementary figure S4**

**
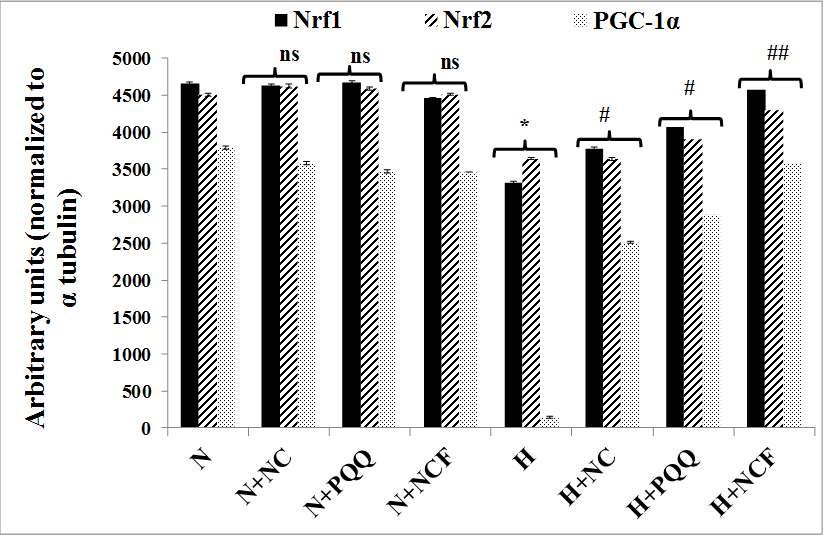
**

**Supplementary figure S5A**

**
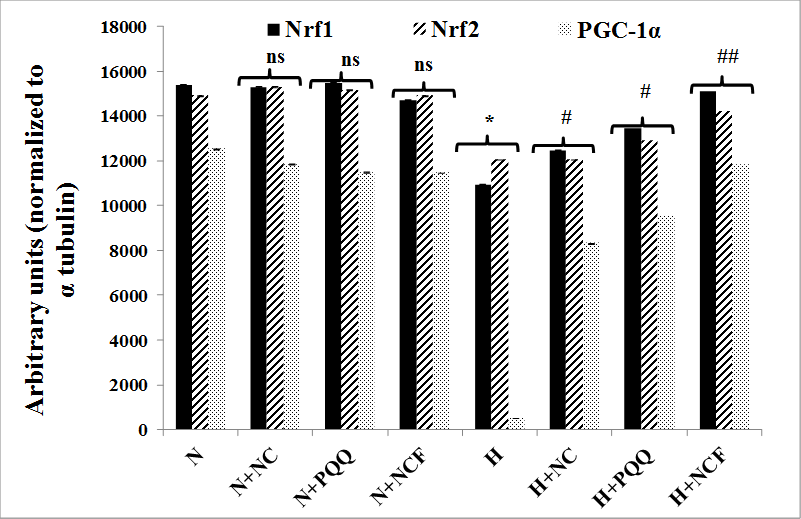
**

**Supplementary figure 5B**

**
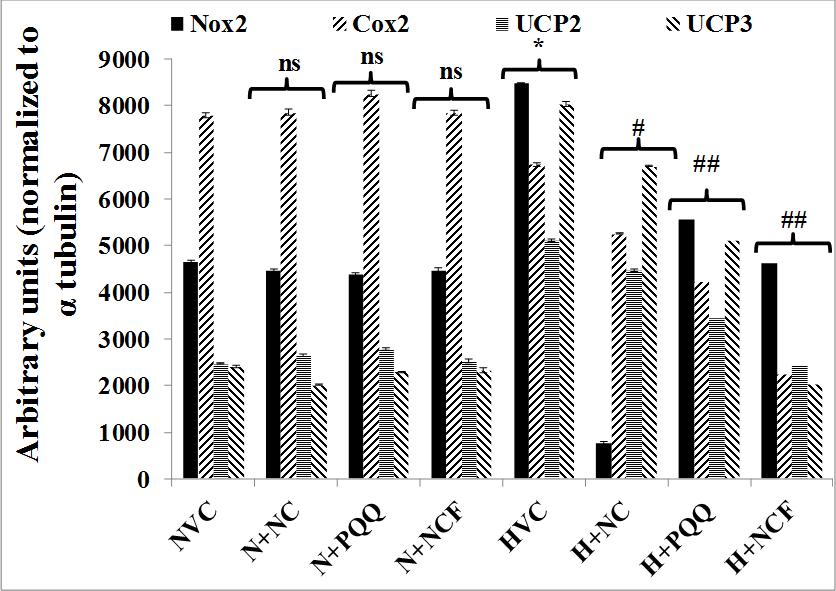
**

**Supplementary figure 5C**

**
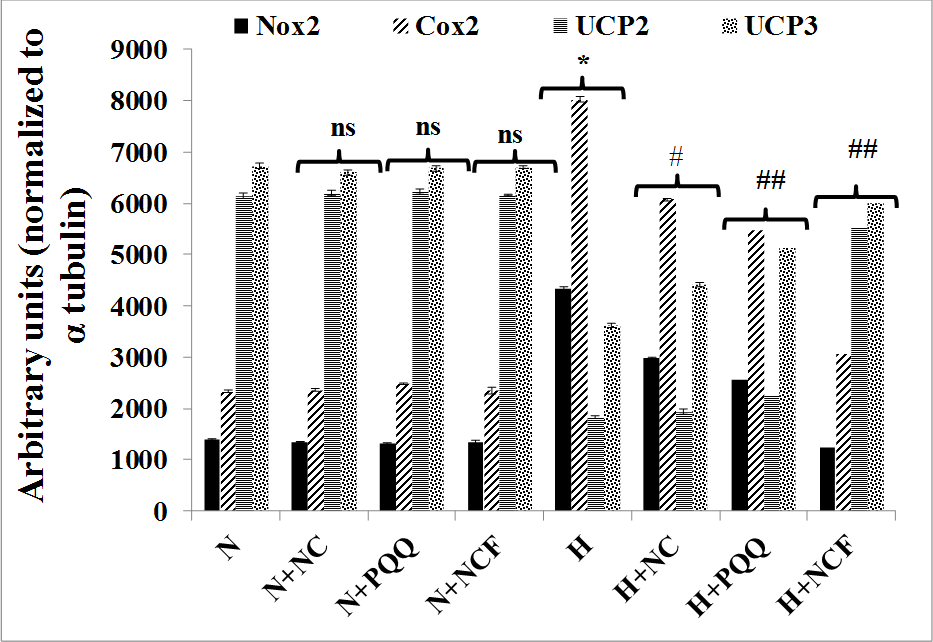
**

**Supplementary figure 5D**

**
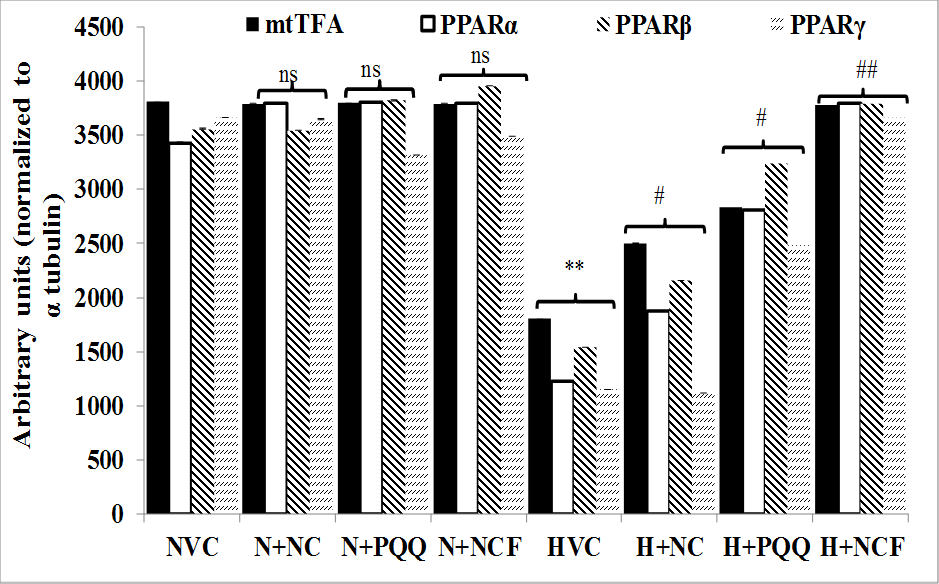
**

**Supplementary figure 5E**

**
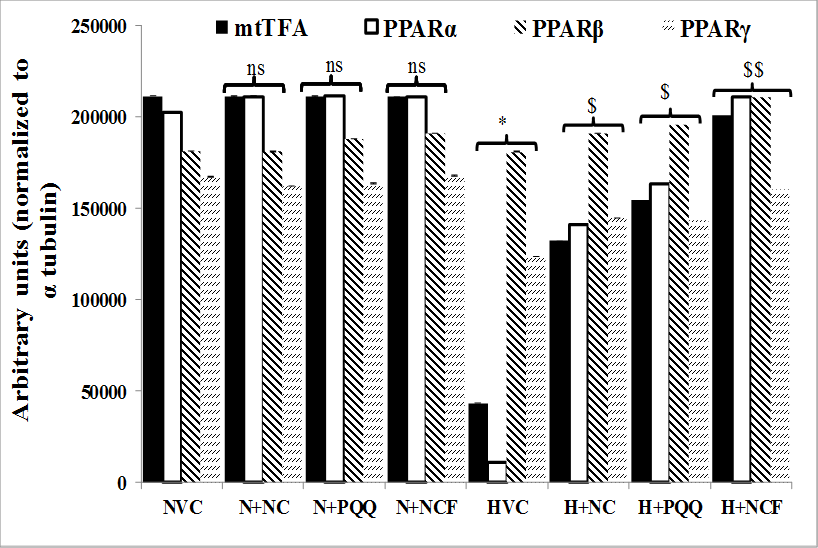
**

**Supplementary figure 5F**

**
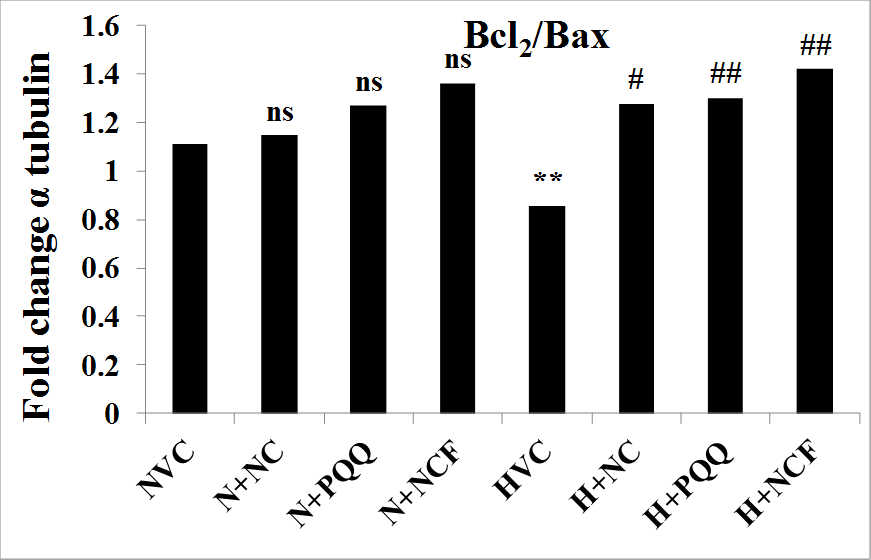
**

**Supplementary figure 5G**

**
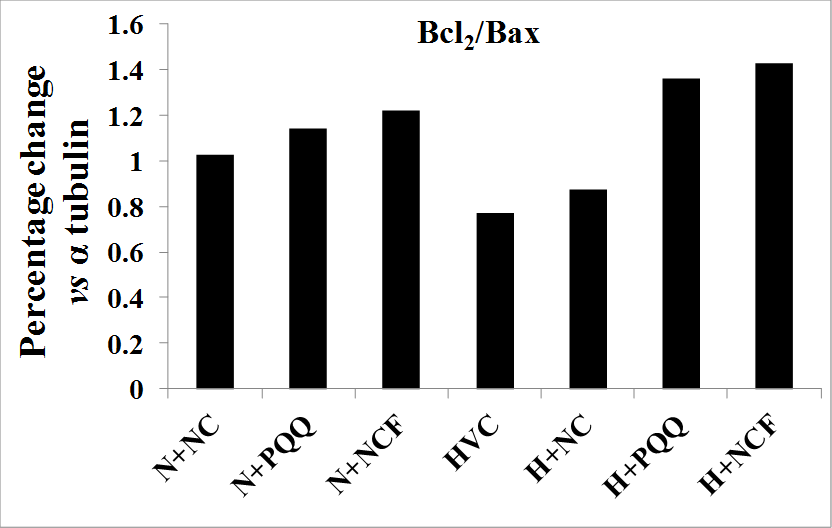
**

**Supplementary figure 5H**

**
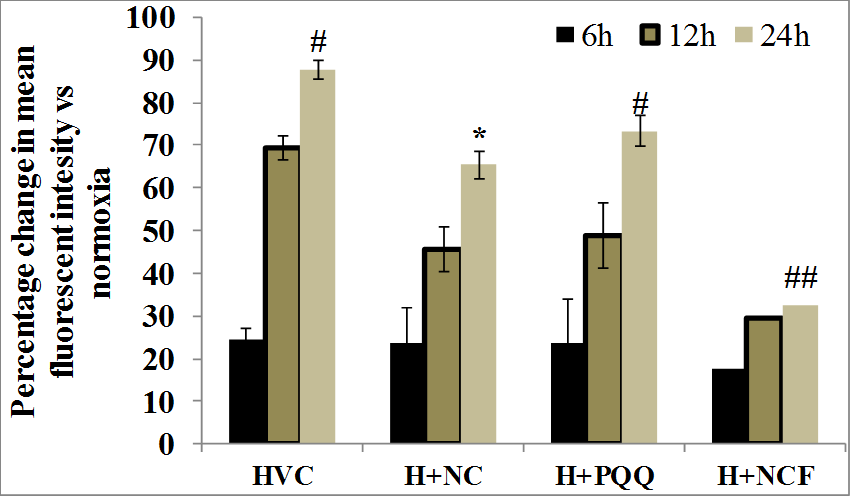
**

**Supplementary figure S6**

**
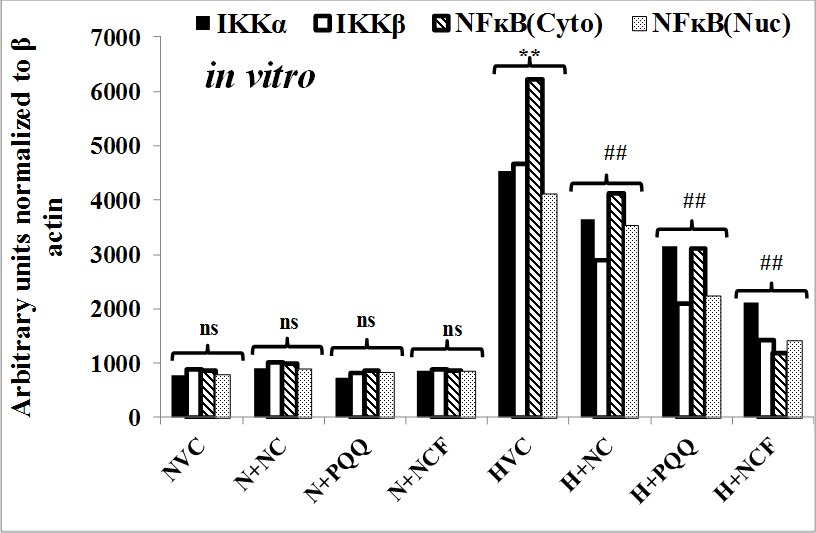
**

**Supplementary figure S7**

**
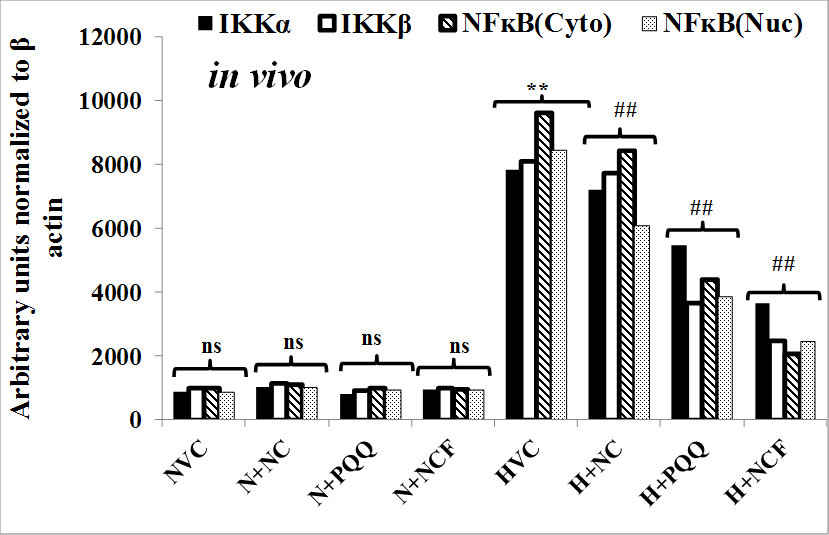
**

**Supplementary figure S8**

| **S.NO** | **GENE** | **Forward** | **Reverse** | **sequence id** |
| --- | --- | --- | --- | --- |
| 1 | *NRF1* | TCGTCTGGATGGTCATTTCA | AGCCCATCTCGTACCATCAC | NC_005103.3 |
| 2 | *NRF2* | TCGTCTGGATGGTCATTTCA | AGCCCATCTCGTACCATCAC | NC_005103.3 |
| 3 | *PGC-1α* | CACGAGGAAAGGAAGACTAA | TAACTCTCGGCCCACCTTACACG | NC_005113.4 |
| 4 | *NOX-2* | CTTGAAGCTTGTGGTCTGCTT | TCTGGACCCCACTTCAAACC | NC_005120 |
| 5 | *COX-2* | TCCATTTGTGAAGATTCCTGTGTT | TTGCTGGCTACCACTCACAC | S67722.1 |
| 6 | *UCP-2* | CGTCTGCACTCCTGTGTTCT | CAATACAGGCTGCTGTCCCA | AB010743.1 |
| 7 | *UCP-3* | GGCACGAGAGTGAATGGTGA | GGAGCGTTCATGTATCGGGT | U92069.1 |
| 8 | *mtTFA* | CAAGGGACTTCGTTCGTCCA | AGAAGGCGACAGAAAGTGGG | AF264733.1 |
| 9 | *PPAR alpha* | GGCCTTGACCTTGTTCATGT | TCACACAATGCAATCCGTTT | NM_013196.1 |
| 10 | *PPAR beta* | CCTAGCTGGCCACAACAAGAA | GAGGAGACTCTGGGTACTGGG | NC_005102.4 |
| 11 | *PPAR gamma* | GGAGCCGTGACCACTGACA | TGGTTTGCTGCATGGTTCTG' | NC_005103.3 |
| 12 | *Bcl-2* | GCTACGAGTGGGATACTGG | GTGTGCAGATGCCGGTTCA | NC_005100.4 |
| 13 | *Bax* | GCTACGAGTGGGATACTGG | GTGTGCAGATGCCGGTTCA | NM_017059 |
| 14 | *MMP2* | GGTGGCAATGGAGATGGACA | CCCGGTCATAATCCTCGGTG | NM_031054 |
| 15 | *MMP9* | GATCCCCAGAGCGTTACTCG | GTTGTGGAAACTCACACGCC | NM_031055.1 |
| 16 | *Col1a1* | AAGGCTCCCCTGGAAGAGAT | CAGGATCGGAACCTTCGCTT | NM_053304.1 |
| 17 | *Col3a1* | TTCCTGGGAGAAATGGCGAC | ACCAGCTGGGCCTTTGATAC | NM_032085.1 |
| 18 | *Alpha tubulin* | TCTGCTTCCCAGGTCTGAAC | TCTGTATTTGGGTGCGTCCC | NC_005120.3 |

**Supplementary table T1**

| **Parameter** | **NVC**  **(Mean±SD)** | **N+NCF (Mean±SD)** |
| --- | --- | --- |
| **WBC (M/mm^3^)** | 10.2±0.13 | 8.84±0.49 |
| **Lymphocytes (M/mm^3^)** | 184.4±3.22 | 151.4±5.65 |
| **Monocytes (M/mm^3^)** | 8.8±2.12 | 9.4±1.69 |
| **Granulocytes (M/mm^3^)** | 6.8±1.34 | 39.2±3.95 |
| **RBC (M/mm3)** | 8.4±0.14 | 10.33±1.54 |
| **MCV (fl)** | 119.6±6.47 | 135.5±6.08 |
| **Hct** | 50.2±3.11 | 71.5±5.5 |
| **MCH (pg)** | 39±2.04 | 44.9±6.08 |
| **MCHC (g/dl)** | 65.2±0.28 | 66.3±4.38 |
| **RDW** | 50.4±1.2 | 49.5±1.83 |
| **THR (M/mm^3^)** | 376±6.33 | 317±12.2 |
| **MPV** | 14.2±3.01 | 16.8±0.84 |
| **Pct** | 0.26±0.04 | 0.26±0.11 |
| **PDW** | 14±0.1 | 16.9±2.14 |

**Supplementary table T2**

| **Parameter** | **NVC**  **(Mean±SD)** | **HVC**  **(Mean±SD)** |
| --- | --- | --- |
| **pH** | 7.372±0.04 | 7.2425±0.04 |
| **PCO_2_ (mmHg)** | 46.1±2.11 | 45.125±1.68 |
| **PO_2_( mmHg)** | 50±2.36 | 44±2.31 |
| **Lac (mmol/L)** | 1.36±0.02 | 3.2±1.89 |
| **HC0_3_(mmol/L)** | 26.8±0.14 | 19.575±0.42 |
| **TCO_2_ (mmHg)** | 28±2.62 | 21±4.69 |
| **BE(mmol/L)** | -10±0.14 | -7.75±4.89 |
| **sO_2_(%)** | 94±2.11 | 63.75±3.64 |

**Supplementary table T3**

| **Parameter** | **NVC**  **(Mean±SD)** | **N+NCF**  **(Mean±SD)** |
| --- | --- | --- |
| **Na (mmol/L)** | 140.63±6.33 | 142.75±8.05 |
| **K(mmol/L)** | 6.334±0.56 | 6.325±1.82 |
| **Cl (mmol/L)** | 107.17±2.01 | 110.75±1.70 |
| **iCa(mmol/L)** | 1.08±0.35 | 0.9075±0.605 |
| **Glu (mmol/L)** | 144±3.11 | 139.5±2.48 |
| **BUN/Urea (mmol/L)** | 17±5.14 | 19.25±7.22 |
| **TCO_2_ (mg/dl)** | 25±3.6 | 23.5±4.2 |
| **Crea** | 0.29±0.089 | 0.35±0.129 |
| **Hct (%)** | 42.17±2.36 | 48.75±3.77 |
| **AnGap (g/dl)** | 21±2.3 | 19.5±1.13 |
| **Hb (g/dl)** | 14.3±0.34 | 19.025±0.94 |

**Supplementary table T4**
